# Plasmon assisted synthesis of TiN-supported single-atom nickel catalysts

**DOI:** 10.1186/s11671-024-03992-z

**Published:** 2024-03-19

**Authors:** Keeniya-Gamalage-Gehan Chaturanga De Silva, Naomi Helsel, Hirithya Sharad Jeyashangararaj, Pabitra Choudhury, Sanchari Chowdhury

**Affiliations:** 1 Department of Chemistry, New Mexico Institute of Mining and Technology, Socorro, NM 87801 USA; 2 Department of Chemical Engineering, New Mexico Institute of Mining and Technology, Socorro, NM 87801 USA

**Keywords:** Single atom, Catalyst, Nickel, Plasmonic, Titanium nitride, Photodeposition

## Abstract

We report the deposition of single atom nickel catalyst on refractory plasmonic titanium nitride (TiN) nanomaterials supports using the wet synthesis method under visible light irradiation. TiN nanoparticles efficiently absorb visible light to generate photoexcited electrons and holes. Photoexcited electrons reduce nickel precursor to deposit Ni atoms on TiN nanoparticles’ surface. The generated hot holes are scavenged by the methanol. We studied the Ni deposition on TiN nanoparticles by varying light intensity, light exposure time, and metal precursor concentration. These studies confirmed the photodeposition method is driven by hot electrons and helped us to find optimum synthesis conditions for single atoms deposition. We characterized the nanocatalysts using high-angle annular dark-field scanning transmission electron microscopy (HAADF-STEM), energy dispersive X-ray spectroscopy (EDX), and X-ray photoelectron spectroscopy (XPS). We used density functional theory (DFT) calculations to predict favorable deposition sites and aggregation energy of Ni atoms on TiN. Surface defect sites of TiN are most favorable for single nickel atoms depositions. Interestingly, the oxygen sites on native surface oxide layer of TiN also exhibit strong binding with the single Ni atoms. Plasmon enhanced synthesis method can facilitate photodeposition of single atom catalysts on a wide class of metallic supports with plasmonic properties.

**Supplementary Information:**

The online version contains supplementary material available at 10.1186/s11671-024-03992-z.

## Introduction

Single-atom catalysts (SACs) have attracted extensive attention due to their high atom efficiency and excellent catalytic performance. Water–gas shift reaction, Fischer–Tropsch synthesis, and alkynes selective hydrogenation, CO oxidation, CO_2_ reduction, oxygen reduction reaction, NO reduction and hydrocarbon oxidation are very few examples of that [1]. SACs possess not only 100% atom efficiency, but also possess high activity due to the low-coordination environment of metal centres in the small HOMO–LUMO bandgap. There are several methods proposed for single atom catalysts synthesis including bottom-up strategies and top-down approach. The top-down strategies such as high-temperature atom trapping approach and in-situ pyrolysis methods can afford single atom catalysts with precise structure and high loading and yield. However, these methods require extremely high temperature limiting their wide scale applications. Mass-selected soft-landing techniques, atomic layer deposition (ALD), and wet chemical routes, are some common bottom-up synthesis methods for single atoms catalysts. Among these methods, the wet chemical method is advantageous for large scale applications as this method doesn’t require specialized equipment and can be performed in wet chemistry lab [1–8]. Recently, photoreduction of metal salts on photocatalytic substrate such as TiO_2_ is demonstrated as an effective method to prepare relatively high loading stable single atom catalysts [9, 10]. Photochemical synthesis of single atom catalysts using wet chemical method where the reduction step can be done using light eliminating the need of high temperature, has potential to implement in the industry scale. It provides the advantages of photo-induced processing, that is, clean process, high spatial resolution, and convenient useful. However, this method requires the support to be photocatalytic limiting the choice of substrates to be semiconducting materials. Here for the first time, we deposit a single atom of nickel catalyst by reducing metal precursor salts with the photo-excited electrons generated on plasmonic nanomaterials. Plasmonic nanoparticles sustain strong charge oscillations when Illuminated with the right wavelength, which are commonly refer to as plasmons. These excitations interact strongly with light and produce a large concentration of the electromagnetic field in nanoscale volumes. After excitation, plasmons can decay following two different pathways: (i) remitting light as scattering, or (ii) absorbing light to generate a transient population of non-equilibrium (hot) charge-carriers that eventually release their energy to the structure in the form of heat, thus increasing the particle temperature. The photoexcited carriers can be transferred to reduce the adsorbed metal precursor when there is a favorable energetic alignment among them [11]. Our recently published paper on visible light mediated plasmon enhanced deposition of nickel and platinum nanoparticles on titanium nitride nanomaterials provides the evidence of that. [11]

In addition to providing all the advantages of photochemical synthesis method, plasmon enhanced deposition method can extend the advantages of photodeposition to wide class of metallic supports with high conductivity. Additionally, it offers several other advantages: (i) concentrating electromagnetic field in the nanoscale, hence can drive precursor reduction reaction right on the substrate surface; (ii) visible light induced reactions providing opportunity to develop solar light enhanced processes; (iii) photothermal effects of plasmonic nanomaterials provide additional pathways to improve metal precursor reductions; (iv) the wavelength and intensity of absorbed light as well as hot electron generation efficiency of plasmonic nanoparticles can be optimized by tuning their shapes and size; (v) solution phase and single pot synthesis can be easily implemented in any wet lab.

Here, we present a combined experimental and theoretical study to deposit single atom nickel catalysts on refractory plasmonic titanium nitride supports. Transition metal nitride nanomaterials such as titanium nitride were considered as promising candidates for the support materials for transition metal catalysts due to their good electrical conductivity and its high resistance to corrosion and acid environment [12–17]. Due to their plasmonic properties they can efficiently absorb and concentrate light at the nanoscale to generate intense electromagnetic fields [18, 19]. The photoexcited electrons can undergo nonradiative decay, generating highly energetic electrons. These photoexcited electrons can reduce metal precursors adsorbed on the surface to deposit metal atoms on the surface [11]. Titanium nitride (TiN) displays tunable stoichiometry of Ti and N and also presence of oxygen on the surface, which can provide favourable binding sites for single atoms. TiN has been identified as a promising candidate for catalyst support for different metal catalysts including Ni, Pt and Co [12, 13, 20, 21]. Deposition of single atom transition metal catalysts such as Ni and Pt on titanium nitride is particularly advantageous due to their strong electronic interaction with the supported transition metal catalysts. TiN supported catalysts have shown significant promise for direct hydrogen and methanol fuel cells, electrocatalytic oxidation of small organic molecules and electrochemical oxygen reduction reactions [22]. In particular, titanium nitride supported nickel catalysts exhibited enhancement in different important reactions including hydrogenolysis of aryl ether, dry reforming of methane, photoreduction of bicarbonate. [11, 22–24]

To understand the mechanism of plasmon-enhanced deposition, we studied the Ni deposition on TiN nanoparticles by varying the synthetic conditions such as light intensity, light exposure time and metal precursor concentration. The understanding developed from the studies helped us to achieve optimum conditions for Ni single-atom catalysts depositions on TiN nanoparticles. We used Density Functional Theory (DFT) based calculations to understand interactions of Ni atoms with the anchoring surface atoms of TiN to predict the favourable deposition sites and stability as well as their electronic interactions. The outcome of this study will guide the synthesis of single atom catalysts on wide range of catalytic support materials including many metals and few transition metal nitrides and carbides, chalcogenide, and oxide nanostructures, which exhibit plasmonic properties.

## Experimental methods

### Materials

TiN nano-cubes with an average size of 20 nm were purchased from US Research Nanomaterials, Inc. (USA). 36.5–38% Hydrochloric acid was purchased from VWR CHEMICALS (USA). 98% Nickel (ll) chloride hexahydrate was purchased from Alfa Aesar (United Kingdom). 99.5 + % anhydrous ethanol was purchased from ACROS ORGANICS (Canada). Anhydrous methyl alcohol was purchased from MACRON FINE CHEMICALS.

### Synthesis of TiN_Ni nanocomposites

400 mg of TiN nanoparticles dispersed in 20 mL of 35% HCl solution was sonicated for 10 min at room temperature in a sealed container. The above sonicated solution was then kept in a water bath at 65 °C for 1 h under a continuous N_2_ (g) flow. After 1 h of heating, the mixture was filtered and washed with 1:1 water: ethanol solution. Again, it was washed twice with 40 mL of anhydrous ethanol. Obtained TiN was concentrated into 5 mL of ethanol. Then the above concentrate was dried in an oven at 80 °C overnight. [11]

A 1.6 mM TiN solution was prepared in 50 mL of 1:1 (v/v) water: methanol solution. The above solution was sonicated for 10 min. Then NiCl_2_ (aq) was introduced to the above TiN solution where the final concentration of NiCl_2_ (aq) is varied. This mixture was then stirred and illuminated with different wavelengths and intensities of light for different durations. An LED light source (X-Cite TURBO XT600-T LED, Canada) is used to illuminate the reaction mixtures with different wavelength light. A Fiber Lite DC-950 Halogen lamp (made in the USA) is used as a broad-spectrum white light source. At the end of the reaction, the reaction mixture was centrifuged and rinsed twice with deionized water to collect the nanoparticles in a solid form. During this study the light source, light intensity, Ni precursor mole fraction with respect to TiN, and the light irradiation time were systematically varied to study their effects.

### Kinetics studies

Kinetics studies were carried out by studying the consumption of methanol in the system during the redox reaction by monitoring the IR peak for the C–O bond of methanol over time. The reaction mixtures were prepared as mentioned above. The main factors studied using kinetics studies are the impact of light intensity and the Ni^2+^ mol fraction on the reduction rate of Ni^2+^. During the light intensity variation studies, a halogen lamp (Fiber Lite DC-950) was placed aside from the FTIR spectrophotometer (Thermo Scientific Nicolet iS50 FT-IR). ~ 1 mL of the reaction mixture was placed on the sample holder. Using a Teflon ring, the liquid sample was stabilized on the detector. Additionally, a fused-silica slide was placed over the liquid reaction mixture to prevent evaporation. Then using a Fresnel lens and a 90° off-axis parabolic mirror (50.8 mm Dia. × 101.6 mm EFL, UV Enhanced Aluminium 50 Å 90° Off-Axis Mirror, Edmund Optics), the light beam from the halogen lamp was focused on to the sample mounted on the FTIR spectrophotometer. The data collection was performed every 34 s.

### Characterization

Nanoparticles were characterized by UV–VIS spectroscopy for their extinction properties (Agilent Technologies: Cary 60, Malaysia), and by X-ray diffraction for their compositional information (X'Pert Pro XRD). They were further characterized with high-resolution transmission electron microscopy (HRTEM), scanning tunnelling electron microscopy (STEM), and energy-dispersive X-ray spectroscopy (EDS) using JEOL 2010 FEG STEM with Oxford EDS and JEOL Neo ARM 200CF TEM equipped with aberration correlation and Oxford Aztec EDS. X-ray photoelectron spectroscopy (XPS) measurements were taken using a Kratos AXIS ULTRA X-ray Photoelectron Spectrometer. XPS samples were prepared by drying the samples at 90 °C for 1 h and being collected in a powdered form. The samples were scanned, resulting in a full range scan as well as a high-resolution scan for Nitrogen, Oxygen, Titanium, and Nickel. Raw response data from the XPS were normalized via an assumption of summations between normal distributions and matched to existing libraries of binding energies. The Ni loadings on TiN were determined by inductively coupled plasma spectrometry-mass spectrometry (ICP-MS) using Agilent ICP-MS 7900. The samples for ICP-MS were prepared by drying the sample at 70 °C for 8 h under vacuum, the collected dry powder sample was then dispersed in 1% nitric acid solution at the concentration of 1 mg/L.

### Computational methods

The Vienna Ab Initio Simulation Package (VASP) was used to carry out spin polarized density functional theory (DFT) calculations [25–27]. The interactions between valence electrons and frozen cores were described by a 400 eV energy cut-off and the Perdew-Burke-Ernzerhof (PBE) form of the generalized gradient approximation (GGA) [28] of the projected-augmented wave method (PAW) [29, 30]. In all of our calculations, Van der Waal interactions were accounted for by a semi-empirical scheme proposed by Grimme (DFT-D2) [30]. The Gaussian smearing method with a width of 0.05 eV around the Fermi level was used to facilitate convergence. The electronic energies were converged to 10^–6^ eV and the ionic relaxations had a tolerance of 0.02 eV/Å for the residual forces on the atoms. The cell was periodic in the x and y directions. To ensure negligible interactions between periodic images, the z dimension was chosen to be 25 Å. The Brillouin zone was sampled using a 3 × 3 × 1 Monkhorst–Pack *k*-point mesh.

Comprised of 50 titanium and 50 nitrogen atoms, the titanium nitride (TiN) cell was 14.97 × 14.97 Å in the x and y directions (Fig. SI.1). This was chosen to represent a pristine TiN that was not oxidized. Same cell as TiN was used to simulate Ti_x_O_y_N_z_ with the exception that there was a single oxygen substitution in place of a nitrogen in the TiN substrate to represent a partially oxidized surface (Fig. SI.2). The anatase titanium dioxide (TiO_2_) cell was 15.23 × 15.23 Å in the x and y directions and comprised of 64 titanium and 128 oxygen atoms (Fig. SI.3). This was chosen to represent a fully oxidized surface of TiN.

## Results and discussion

### Characterization of TiN

**Fig. 1:**
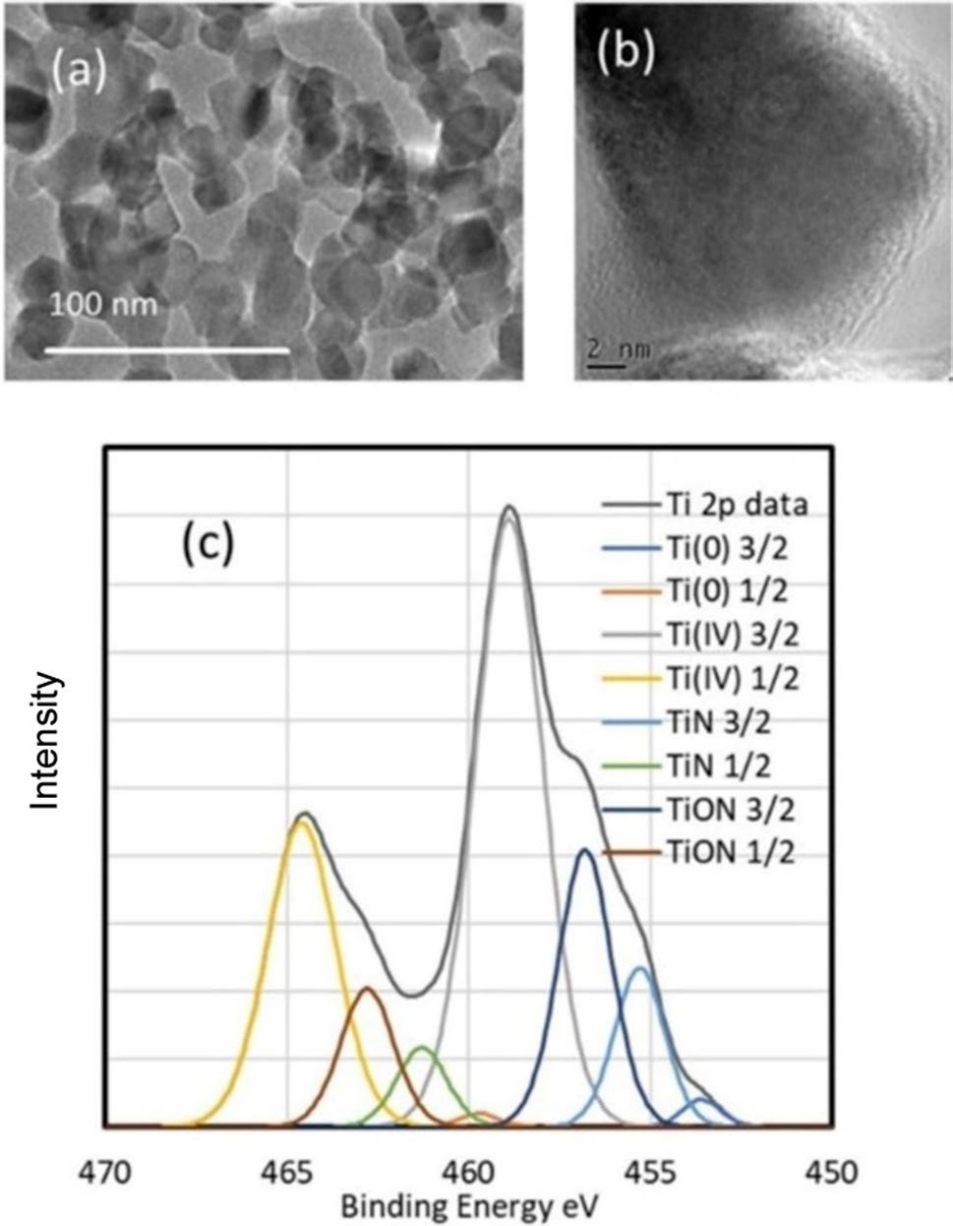
**a** HRTEM of TiN nanoparticles **b** magnified image to show amorphous oxide layer on the surface of TiN nanoparticles. **c** XPS Ti 2p data analysis of TiN samples

The morphology of TiN nanoparticles characterized using high-resolution transmission electron microscopy (HRTEM) as shown in Fig. 1. It is well known that TiN nanoparticles develop 1–2 nm thick self-passivating oxide layer on the surface (Fig. 1b). However, as shown in the previous papers from our group, this thin oxide layer has negligible effect on the optical properties TiN nanoparticles [31, 32]. High resolution TEM image of TiN nanoparticles as shown in Fig. 1b indicates that the maximum thickness of the oxide layer on the surface of TiN nanoparticles is 2 nm. To understand the effect of oxide layer on the optical properties, we calculated absorption spectra of 20 nm TiN nanoparticle with different thickness of oxide layer using discrete dipole approximation method. As shown in Fig. SI.4, the 2 nm thick oxide layer on the surface of TiN nanoparticles doesn’t significantly influence it’s optical properties. The negligible effect of oxide layer is also evident by the UV–Vis absorbance peak of TiN nanoparticles which show usual plasmonic peak around 650 nm (Fig. 3a). However, our calculation suggests increasing the thickness of oxide layer gradually decreases the light absorbance of TiN nanoparticles and redshift its light absorption peak. We can see a significant decrease in light absorption when the oxide layer thickness reaches 10 nm. XPS analysis confirmed that the surface layer is a mixture of TiN, TiO_2_ and titanium oxynitrides (Ti_x_O_y_N_z_) (Fig. 1c and Fig. SI.5). Based on the Ti 2p high resolution XPS data for the nanoparticles, the nanoparticle surfaces are comprised of ~ 18% TiN, ~ 27% Ti_x_O_y_N_z_ and ~ 55% TiO_2_. Similarly, the high resolution N 1 s XPS spectra of TiN_Ni indicate the presence of oxide layer on the surface. This is interesting as it provides different anchoring surfaces for Ni atoms. Additionally, formation of surface oxide layer on TiN can improve the lifetime of the hot electrons making it easier to harvest them for hot electron mediated reduction reactions [33]. Fig. SI.6 depicts the x-ray diffractograms (XRD) data for TiN. TiN mostly shows the rock-salt crystal structure of osbornite.

### Deposition of Ni on TiN

**Fig. 2:**
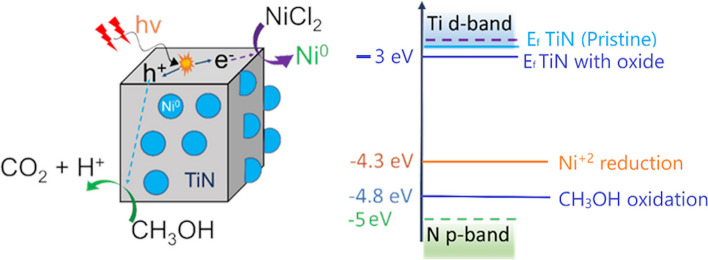
**a** Schematic of the plasmonically driven photodeposition of Ni on TiN nanoparticles **b** Energy levels associated with Ni photodeposition on TiN nanoparticles

Titanium nitride nanomaterials can strongly absorb broad spectrum solar light. Under visible light irradiation TiN can generate hot electrons both due to interband and intraband transitions as its interband transition (N p-band to Ti d-band) is ~ 2 eV (Fig. 2) [34]. One important factor for photodeposition of metals on support materials is the favorable positioning of the energy bands and the potential values. Interestingly, the presence of oxide layer on the surface of titanium nitride nanoparticles can increase the work function. For example, while the work function for pristine stoichiometric TiN is found to be 2.6 eV, the presence of oxide layer on the surface can increase the work function to 3 eV. [32] However, even with the presence of oxygen the Fermi energy level of TiN is such that the reduction of Ni precursor is energerically favorable both for the excited electrons generated from interband and intraband transitions (Fig. 2b). However, methanol can mostly scavenge the holes in N p-band mainly generated by the interband transitions.

**Fig. 3:**
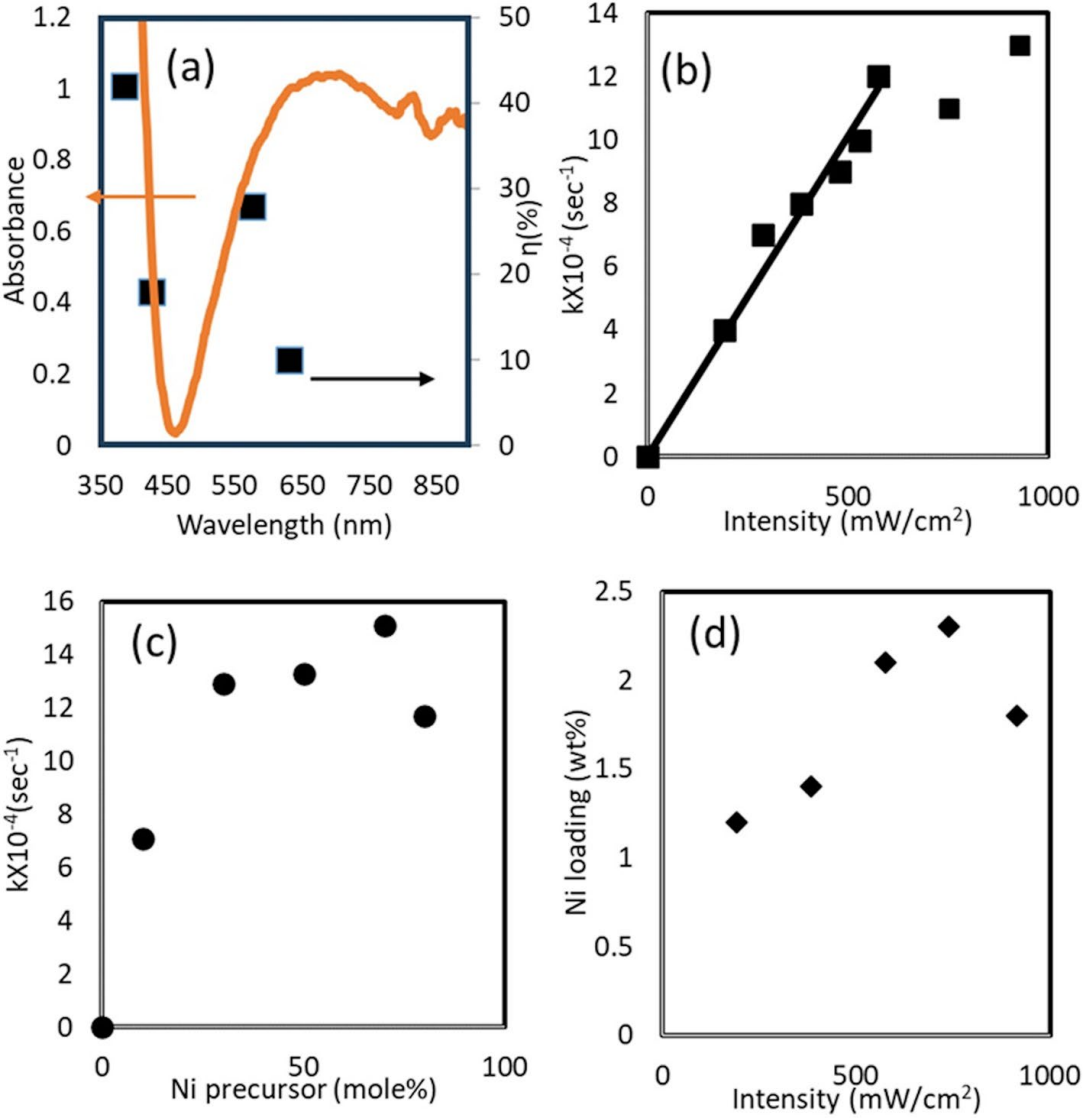
**a** Action spectra with TiN extinction spectra for TiN_Ni synthesis at 95.4 mW cm^−2^ illumination light intensities **b** Methanol consumption rate on different intensities of broad-spectrum halogen light. **c** Dependence of methanol consumption rates on mole fraction of Ni precursor. **d** Ni loading (wt%) on TiN nanostructures as determined by ICP-MS

An experiment with in situ FTIR studies of reactions under light is developed to study the rate of the consumption of methanol in the reaction mixture by monitoring the depletion of the FTIR peak for methanol C–O bond at ~ 1000 cm^−1^ (See SI for the detailed information, Fig. SI.7). While direct reduction of metal cations could be challenging to study with FTIR, the consumption of methanol is used to indirectly probe the Ni atom depostion rate via Ni precursor reduction (Please see SI for the details of these experiments). We studied the effect of different parameters such as light wavelengths, light intensity, and the metal precursor concentrations on the reaction. The purpose of these studies were two fold. First to unravel the mechanism of plasmon enhanced deposition and second to understand the effect of above parameters on Ni atom deposition rate to find an optimum condition for single atom deposition. The deposition of Ni on TiN only occurred in the presence of light. Additionally, there was no formation of Ni nanostructures in the absence of TiN. These confirm that the Ni deposition process is light mediated and driven by plasmonic properties of TiN. Several possible contributions for plasmonic enhanced reactions are enhanced near-fields, photoexcited charge carriers transfer and local photothermal heating [35]. It’s important to unravel the particular mechanism which enhance the plasmon enhanced deposition to fully exploit the advantages of such method. First, we pursued an wavelength dependent studies to confirm that the deposition of Ni on TiN is indeed due to the reduction of Ni precursors by photoexcited charge carriers generated on TiN (Fig. 3a).

We studied the photonic efficiency (ɳ) of the reaction at different excitation wavelength to understand the effect of photoexcited electrons generated from interband or intraband transition on Ni deposition. Photonic efficiency (ɳ) is the ratio between rate of the reaction of interest (*r*_*i*_) and the photon flux (*ρ*) Eq. (1). Photon flux is the number of photons irradiated on the sample for a unit time.

1 $$\eta =\frac{{r}_{i}}{\rho }$$

2 $$\rho = \frac{{N_{p} }}{{N_{A} }}$$

$$N_{p} :{\text{Number}}\;{\text{of}}\;{\text{photons}}\;{\text{per}}\;{\text{unit}}\;{\text{time}}$$

$${N}_{A}:{\text{Avagadro's}\;\text{constant}}$$

3 $${N}_{p}= \frac{I*A}{{E}_{p}}$$

$${\text{I}}:\mathrm{Irradiance }({\text{mW}}/ {{\text{cm}}}^{2})$$,

$${\text{A}}:{\text{area of illumination}}\,({{\text{cm}}}^{2})$$

$${\text{E}}_{{\text{p}}} :{\text{Energy}}\;{\text{of}}\;{\text{a}}\;{\text{photon}}\;{\text{depending}}\;{\text{on}}\;{\text{its}}\;{\text{wavelength}}\; ({\text{mW}})$$

We found that when irradiating the reactant solution with shorter wavelengths like 385 nm, we see highest photonic efficiency (40%). Following the TiN absorbance spectrum, it goes down to 20% at 450 nm and again increased at 575 nm to 30%. Interestingly at 630 nm, the efficiency is at its minimum (~ 10%) (Fig. 3a). The SPR excitation of TiN nanoparticles results in both intraband and interband transitions.

The wavelengths above 600 nm mostly excite intraband transitions as the interband transition energy of TiN is ~ 2 eV. Both intraband and interband transitions can generate photoexcited electrons suitable to reduce Ni precursors (Fig. 2b). Intraband transition from Fermi energy level can generate higher energy hot electrons; however, the holes generated by this process cannot be scavenged by methanol. Additionally, the recombination rate for excited electrons due to intraband transitions is inherently higher than the electrons generated from the interband transitions [36]. While the formation of surface oxide layer on TiN can improve the lifetime of the hot electrons making it easier to harvest them for hot electron mediated reduction reactions, it may still not be as effective as the electrons generated from interband transitions. This may be the reason for the decreased effectiveness of 630 nm wavelength light to reduce Ni precursors. On the other hand, the interband transition from N p-band to Ti d-band can generate larger population of relatively lower energy electrons as the holes generated in the N p-band can be easily scavenged by methanol.

The wavelength dependency of photonic efficiency of reaction (Fig. 3a) confirms that the reaction is driven by photo-generated hot electrons. It should be noted that in the absence of light we did not observe any evidence of Ni deposition on TiN (Fig. SI.8).

Additionally reaction rate dependency on light intensity is investigated to understand whether the reaction is driven solely by hot electrons or some other factors such as photothermal heat plays in role. (Fig. 3b) The effect of light intensity on reaction kinetics was measured by varying the light intensity of the halogen lamp, while maintaining the Ni precursor mole fraction at 30% with respect to TiN. The methanol concentration obtained from FTIR data vs time plots were fitted for zero order reaction kinetics to extract rate constant. Figure 3b, shows a linear trend for the reaction rate increase with the increase of incident light intensity up to 575 mW/cm^2^ then plateaus. The initial linear trend confirmed the reaction is driven by plasmonically generated hot electrons. The electron hole pair generation rates is directly proportional to the incident light intensity; hence, we see linear increase in reaction rate with the light intensity at the initial stage. However, the recombination rate is also significantly higher at higher intensity and if the hole scavenging rate can not keep up with the recombination rate the effect tapers off [36]. It should be noted that if the reaction is thermally driven, the trend for reaction rates with increasing light intensities should be exponential due to the Arrhenius equation. Additionally, we control the temperature of solution at room temperature using a cooling fan. At the range of light intensities used in our experiments, the difference between the global temperature of the solution and the local temperature of nanoparticles is expected to be negligible [37]. Therefore, the photodeposition can not be due to photothermal effects.

We also study effect of metal precursor concentration on the recation rate. The impact of Ni precursor concentration on kinetics were studied by changing the Ni precursor mole fraction 10%, 30%, 50% and 70% with respect to the amount of TiN, while maintaining the light intensity at a constant value of 576 mW cm^−2^. Figure 3c showed an increase of reaction rate with the increase of Ni^2+^ mol fraction. It confirms that with higher intensities and higher mole fractions of Ni^2+^ precursor it is possible to reach a higher Ni^2+^ reduction rate. This is an important knowledge for optimizing the conditions for single atoms depositions as higher precursor reduction rate favours deposition of single atoms on the supports [4, 5]. We used ICP-MS to confirm the Ni loading on TiN at different intensities. As it can be seen for Fig. 3d, in general Ni loading on TiN increases with the increase in light intensity and then tapers off at the highest intensity 975 mW/cm^2^. The trend matches with what we see for methanol oxidation rate (Fig. 3b). JEOL Neo ARM 200CF TEM equipped with aberration correlation and Oxford Aztec EDS analysis is used to further confirm the Ni deposition on TiN nanoparticles for different samples (Fig. SI.9). EDS mapping of Ni/TiN nanoparticle also confirms Ni deposition on the surface of TiN nanoparticles (Fig. SI.10).

**Fig. 4:**
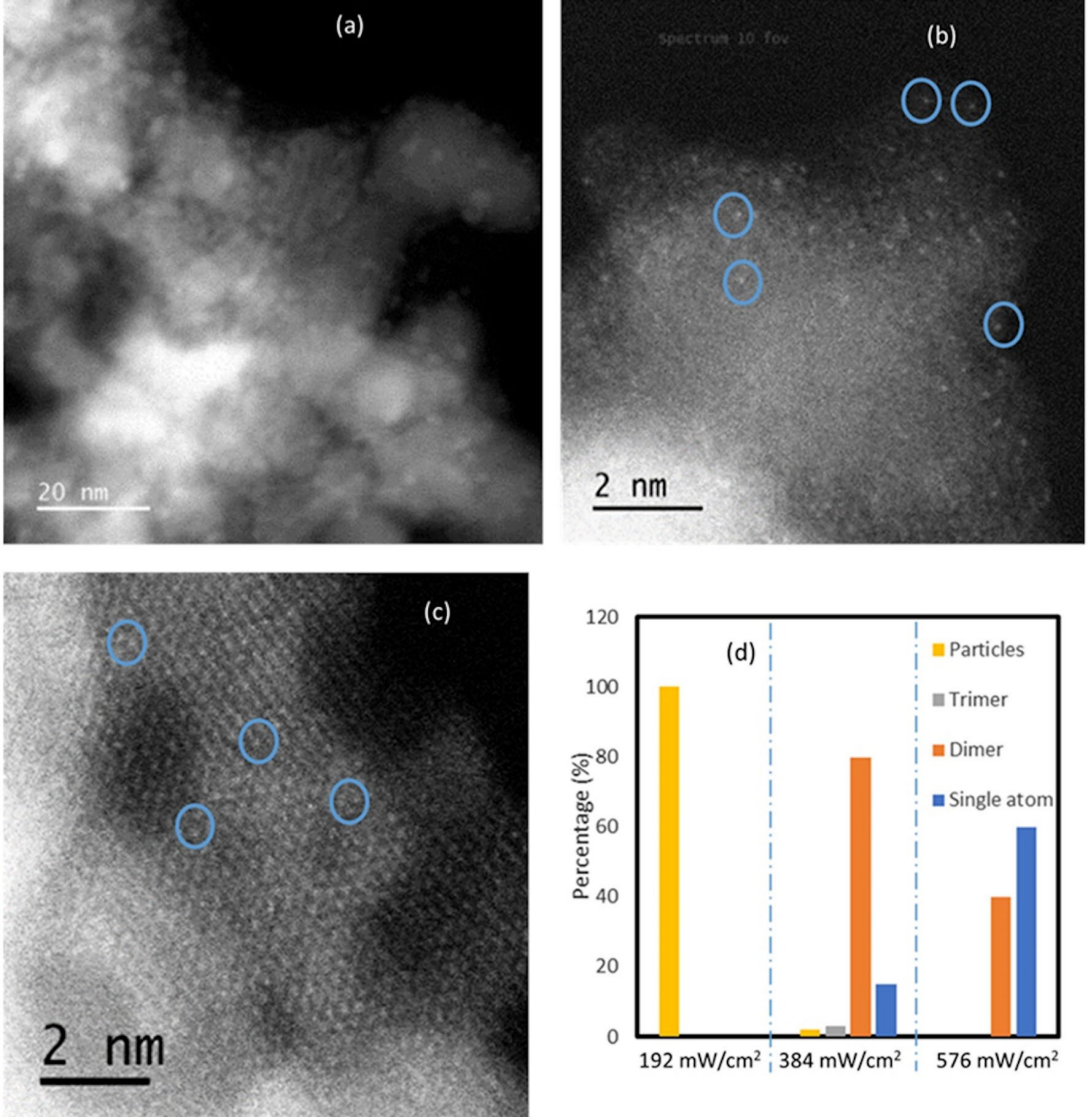
**a–c** show the images collected using High-angle annular dark-field imaging -scanning transmission electron microscope (HAADF-STEM) of Ni/TiN samples deposited under different light intensities of broad-spectrum halogen light after 5 h. **a** 192 mW/cm^2^, **b** 384 mW/cm^2^, and **c** 576 mW/cm^2^. To guide the eye, some of the single atoms are circled in blue. **d** Percentage of deposited Ni single-atoms, atom dimers, atom trimers and particles on TiN under different conditions

Figure 4a–c show the images collected using High-angle annular dark-field imaging -scanning transmission electron microscope (HAADF-STEM) of Ni/TiN samples deposited under different light intensities of broad-spectrum halogen light. The percentage of Ni deposited as single atoms, dimers, trimers, and nanoparticles under different light intensities is determined from the HAADF-STEM images of Ni deposited on TiN (Fig. 4d). We have identified different forms of Ni deposited on TiN by measuring their size using *ImageJ* software. The size of Ni single atoms, dimers and trimers are 0.25 nm, 0.5 nm and 0.75 nm respectively (considering atomic radius of Ni is 0.124 nm). Any particles with the size above that is considered to be nanoparticles.

With the increase of light intensity, we can see a higher percentage of single atoms deposition (Fig. 4b, c and d). This agrees with the fact that higher intensities of light lead to elevated reduction rates of Ni^2+^ precursor enabling more single-atom deposition. Rapid rates of metal precursor reduction at the beginning can introduce a large amount of Ni^0^ available to deposit as single atoms on the favourable sites. This is mainly possible if the cation reduction rate is higher than the nucleation rates of metal atoms on different sites. On the other hand, slower metal precursor reduction rates have given rise to a smaller amount of larger size cluster depositions [38]. To understand the Ni atom deposition on TiN further we have done computational calculations as discussed below.

### Computational calculations

As discussed above the surface layer of TiN nanoparticles is a mixture of TiN, TiO_2_ and titanium oxynitrides (Ti_x_O_y_N_z_), we calculated binding energy of Ni on different sites of pristine TiN, TiO_2_ and TiON and N vacancy sites of TiN.

As mentioned previously, the nanoparticle surfaces were comprised of ~ 18% TiN,  ~ 27% Ti_x_O_y_N_z_, and ~ 55% TiO_2_. It is expected that the nickel will be deposited on all three substrates but be primarily deposited on the TiO_2_. Our calculations suggest multiple nickel stable binding sites are possible on TiN and Ti_x_O_y_N_z_ as well as on TiO_2_ (Table 1, Fig. SI.11, Fig. SI.12). The binding energy tells us the probability of getting single atoms on different sites of TiN/ Ti_x_O_y_N_z_ /TiO_2._ The higher the binding energy of a particular site, the more likely that site is to be present in comparison to its counterparts. As depicted in Table 1, there are three adsorption sites, nitrogen vacancy, nitrogen-top, and titanium top on pristine TiN. We found the nickel on hollow sites and bridge sites are not stable configurations on TiN. The most stable adsorption site on TiN is the nitrogen vacancy site with a binding energy of − 4.98 eV. Meaning that the N-vacancy sites are the most favourable, then the N-top site, and lastly the Ti-top. Because of this, later calculations mainly consider the N-vacancy and N-top sites. The O-top site of the Ti_x_O_y_N_z_ had a binding energy stronger than Ti-top but were still weaker than the N-vacancy and N-top. Interestingly, most of the nickel binding energies on TiO_2_ sites were stronger than the TiN sites. The following sites on TiO_2_ are stable: Ti-bridge, 1-O-bridge, 2-O-bridge, 3-O-bridge, O-vacancy, and Ti-vacancy. The remaining possible sites were tested but they were not stable binding configurations. The O-vacancy site on TiO_2_ had the highest binding energy of − 5.56 eV. The next most stable configuration was the 3-O-bridge site with a binding energy of − 5.33 eV. Both O-vacancy sites and 3-O bridge site on the TiO_2_ show stronger binding energies in comparison to nitrogen vacancy sites on TiN. Since most of the surface of TiN nanoparticles are comprised off TiO_2_, we expect the majority of single atom Ni deposited on the sites of TiO_2_. Although the O-vacancy had the most favourable binding site, we considered the 3-O-bridge site for the possible sites for single atom Ni deposition on TiO_2_ because bridge sites are more abundant on the TiO_2_ surface than O-vacancies or N vacancy sites on TiN.

**Table 1:** Binding energies of Ni on TiN, Ti_x_O_y_N_z_ and TiO_2_ according to adsorption site

Nickel binding site	Binding energy (eV)
N-Vacancy (TiN)	− 4.98
N-top (TiN)	− 3.63
Ti-top (TiN)	− 2.85
O-top (Ti_x_O_y_N_z_)	− 3.13
Ti-bridge (TiO_2_)	− 3.99
1-O-bridge(TiO_2_)	− 5.06
2-O-bridge(TiO_2_)	− 4.60
3-O-bridge(TiO_2_)	− 5.33
O-vacancy(TiO_2_)	− 5.56
Ti-Vacancy(TiO_2_)	− 4.57

#### Prediction of aggregation energies

**Fig. 5:**
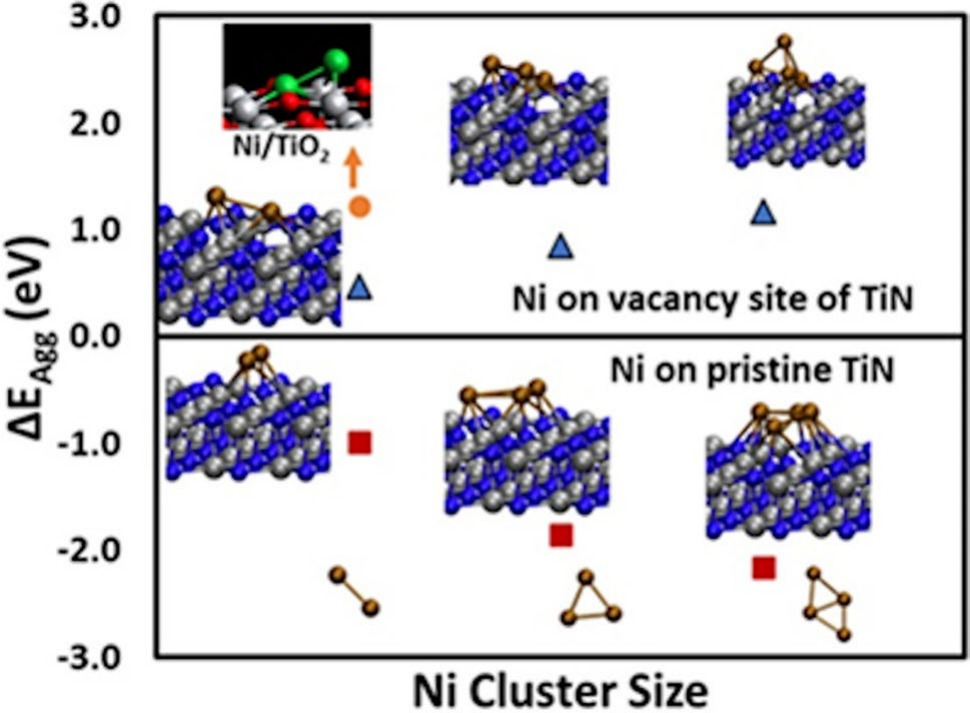
Aggregation energy investigated for formation of surface Ni clusters. Positive values signify the thermodynamic preference for atomically dispersed configurations. Orange circle, red square and blue triangles are for Ni/TiO_2_, Ni/pristine TiN and Ni/defect TiN, respectively

We further calculated aggregation energy of Ni on vacancy sites of TiN, N-top sites of pristine TiN and 3-O-bridge sites of TiO_2_ to predict the possibility of stable single atom deposition [39]. By comparing binding energy of Ni atom at neighbouring stable sites versus isolated sites we evaluated the tendency toward clustering. Aggregation energy is defined as ∆E_agg_=E(n)_total_−(n−1)×E(host)_total_−n×E(SAA)_total_. Our calculations of Ni/TiN and Ni/TiO_2_ (Fig. 5) suggest that Ni single atom can be formed on both vacancy site of TiN and O bridge sites of TiO_2_ surfaces. However, it has more tendency to make clusters on N sites of Pristine TiN. Since more than 50% of the TiN surface is comprised of titanium dioxide, it is safe to assume that most of the single atoms are deposited on O sites of TiO_2_ surfaces.

### XPS analysis

**Fig. 6:**
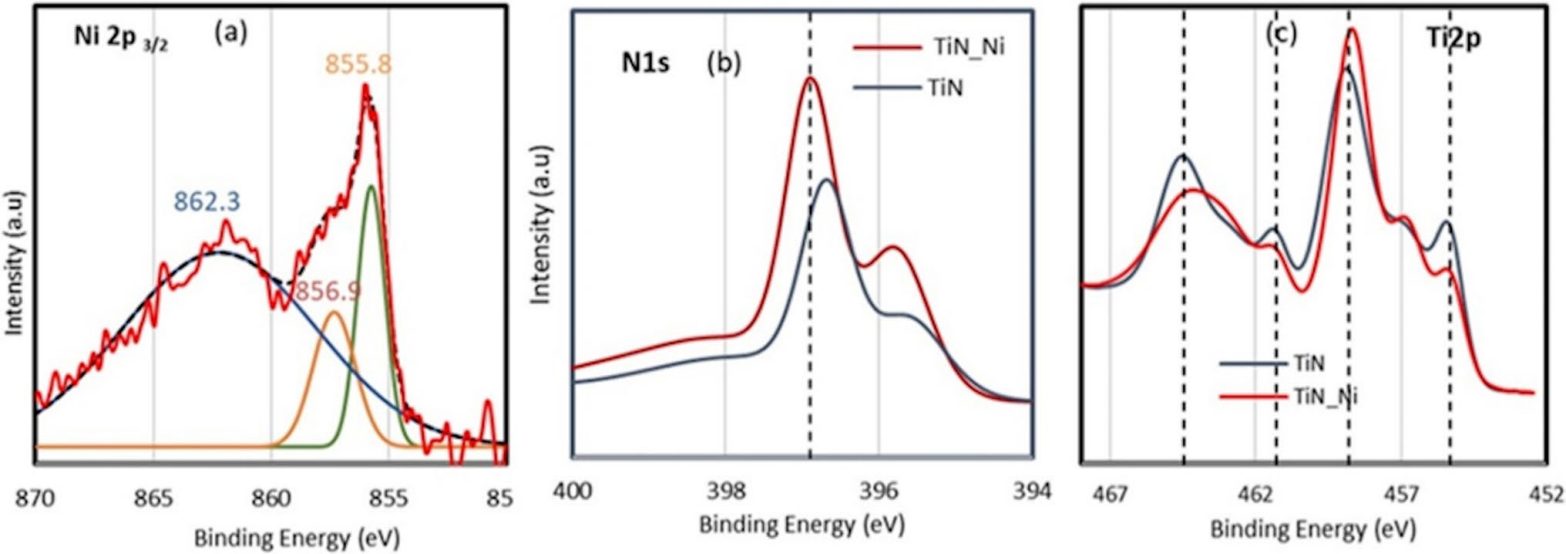
**a** XPS Ni 2p Intensity of nickel deposited on TiN **b** XPS N 1 s peaks comparison among TiN_Ni and TiN samples. **c** Ti 2p peaks comparison among TiN_Ni and TiN samples

XPS analysis is used to understand the chemical state of single atom Ni deposited on TiN. As we can see from Fig. 6 in case of the sample where Ni is mostly deposited as single atoms or dimers, the Ni 2p3/2 XPS spectrum can be deconvoluted to three peaks at 855.8, 856.9, and 862.2 eV respectively. The peak at 862.3 eV can be assigned to satellite peak. The peak at 855.8 is the major peak and the peak at 856.9 is a minor shoulder peak. The Ni 2p3/2 peaks at 855.8 is higher than that of metallic Ni_0_ (B853.0 eV) indicating Ni species are in higher valence state. The major peak observed at 855.8 eV coincides with Ni^3+^ oxidation state [40, 41]. The bonding of oxygen (O) and OH group on Ni can result in more positively charged Ni atoms with high oxidation state as observed by Li et al. [11, 41]This observation indicates most of the single atoms deposited on the oxygen sites of TiN surface. Our calculations as discussed in next section also confirm that the Ni single atom deposited on oxygen sites of TiN surface, shows higher oxidation state with the addition of OH group on it. We also see that the N 1s peak of TiN_Ni sample is shifted to higher binding energy in comparison to TiN alone. That shows N on TiN sample loses electrons upon deposition of Ni on it. The Ti 2p peaks of TiN_Ni sample, especially the one assigned to TiO_2_ (465 and 459 eV peaks) shifts to lower binding energy in comparison to TiN sample, indicating Ti sites gain electrons when Ni deposits on it. Our computation data as discussed below further confirms our XPS data.

### Bader charge analysis

Computational Bader charge analyses were conducted to confirm the validity of the XPS results. Hydroxyl groups were added to the surfaces of the simulated N-vacancy TiN, Ti_x_O_y_N_z_, and TiO_2_ after Ni deposition. Seen in Fig. SI.13, Ni oxidation state increases as the number of hydroxyl groups on the surface increases. Not all hydroxyl groups were observed to be chemically bonded to the Ni atom; however, even the hydroxide groups chemically bonded to the substrate impacted the oxidation state of Ni. This could be why Ni^3+^ is observed instead of the metallic Ni^0^ form. Ni-Ti_x_O_y_N_z_ with four hydroxyl groups was omitted because the Ni desorbs from the surface to form Ni(OH)_4_. The differences of charge of N and Ti were also investigated before and after Ni deposition of N-vacancy TiN, Ti_x_O_y_N_z_, and TiO_2_. Since XPS experiments look at the surface atoms, only the top layer of N and Ti atoms were considered during calculation. A positive charge difference means that there is accumulation of charge on the surface for Ti or N after deposition and a negative charge difference means that there is depletion of charge. The differences of charge of N and Ti investigated before and after Ni deposition of N-vacancy TiN, Ti_x_O_y_N_z_, and TiO_2_ are shown in Table SI1. The surface N atoms consistently showed depletion of electrons, consistent with XPS results. However, the surface Ti atoms were inconsistently showing accumulation or depletion depending on the presence of hydroxide groups and which substrate was used. As shown in Fig. 6c, only some of the Ti XPS peaks show a gain of charge after Ni deposition, which is consistent with our findings. Ti_x_O_y_N_z_ and TiO_2_ varieties both show consistent accumulation of electrons on Ti after Ni deposition, which is consistent with the first and third peaks of Ti *2p* XPS peaks in Fig. 6c. In contrast, N-vacancy TiN does not show a consistent electron accumulation on Ti after Ni deposition. This further indicates that Ni single atom is mostly deposited on titanium oxide sites of TiN surfaces.

## Conclusions

In this work, we have photodeposited Ni on TiN nanoparticles by reducing Ni salt on TiN using the plasmonic properties of TiN. This is significant because Ni is one of the most widely used and cheapest elements in metal-based catalysts and the synthesis of these catalysts often requires high temperatures [42]. In situ FTIR studies under different wavelengths and intensities of light were conducted to understand their effect on the kinetics of photodeposition of Ni atoms. These studies revealed that the photo-excited electrons generated on the TiN is most likely driving the Ni precursor reduction reaction. Increasing the light intensities and molar concentration of Ni precursor, we could improve the Ni precursor reduction rate enhancing the deposition of Ni single atoms on TiN. We achieved the deposition of mostly single atom Ni catalysts on TiN using 576 mW/ cm^2^ intensity of halogen lamp. We could vary the size of Ni catalyst from nanoclusters to single atoms by varying the factors that govern the deposition rate, such as light intensity. XPS studies revealed TiN nanoparticles possess self-passivated oxide layer on the surface comprised of TiN, TiO_2_ and titanium oxynitrides (Ti_x_O_y_N_z_). Using first principles DFT calculations, we found that multiple nickel stable binding sites are possible on pristine TiN, N vacancies on TiN and Ti_x_O_y_N_z_, as well as on TiO_2_. However, Ni atoms are more likely to aggregate and form clusters on pristine TiN. The formation of a Ni single atoms on the N vacancies of TiN and on 3-O-bridge sites of TiO_2_ are energetically favourable as their aggregation energy is positive. Deposition of Ni catalysts on plasmonic supports allow us to develop photo active Ni catalysts as the support can absorb light and transfer the energy or photo-excited charge carriers to catalysts to drive reactions. Additionally, this work is a pioneering step toward using plasmonic properties of support to deposit single-atom catalysts. Synthesis of single atom catalysts using wet chemical method using visible light eliminating the need of high temperature provides the advantages of clean process, high spatial resolution, and convenient useful, hence has potential to implement in the industry scale.

### Supplementary Information

**Additional file 1. **Schematic representations of TiN, Ti_x_O_y_N_z_, and TiO_2_ used for DFT simulations (Fig. SI.1-3). Theoretically calculated absorption efficiency for 20 nm TiN nanoparticles with varying oxide layer surface thickness (Fig. SI.4). N 1S XPS peak and XRD plots of TiN (Fig. SI.5-6). In-situ photodeposition using FTIR experimental setup and results (Fig. SI.7). Explanation of experimental kinetic studies. HAADF-STEM image of control TiN sample with Ni salt in the absence of light (Fig. SI.8). TEM EDS analysis plot and maps confirming Ni deposition on TiN (Fig. SI.9-10). Schematic representations of Ni binding sites on TiN, Ti_x_O_y_N_z_, and TiO_2_ used for DFT simulations (Fig. SI.11-12). DFT calculated nickel oxidation states for increasing OH groups present on TiN, Ti_x_O_y_N_z_, and TiO_2_ surfaces (Fig. SI.13). The DFT-calculated electron differences after deposition for TiN, Ti_x_O_y_N_z_, and TiO_2_(Table SI.1).

## Data Availability

The data that support the findings of this study are available upon reasonable request from the authors.
