# Supplementary material for: Plasmon assisted synthesis of TiN-supported single-atom nickel catalysts
Source: Discov Nano. 2024 Mar 19;19(1):50. doi: 10.1186/s11671-024-03992-z (PMC10951189; doi:10.1186/s11671-024-03992-z)
Supplement: Supplementary file 1 — Additional file 1. Schematic representations of TiN, TixOyNz, and TiO2 used for DFT simulations (Fig. SI.1-3). Theoretically calculated absorption efficiency for 20 nm TiN nanoparticles with varying oxide layer surface thickness (Fig. SI.4). N 1S XPS peak and XRD plots of TiN (Fig. SI.5-6). In-situ photodeposition using FTIR experimental setup and results (Fig. SI.7). Explanation of experimental kinetic studies. HAADF-STEM image of control TiN sample with Ni salt in the absence of light (Fig. SI.8). TEM EDS analysis plot and maps confirming Ni deposition on TiN (Fig. SI.9-10). Schematic representations of Ni binding sites on TiN, TixOyNz, and TiO2 used for DFT simulations (Fig. SI.11-12). DFT calculated nickel oxidation states for increasing OH groups present on TiN, TixOyNz, and TiO2 surfaces (Fig. SI.13). The DFT-calculated electron differences after deposition for TiN, TixOyNz, and TiO2(Table SI.1). [file 11671_2024_3992_MOESM1_ESM.docx]

**Plasmon Assisted Synthesis of TiN-Supported Single-Atom Nickel Catalysts**

**Supporting Information**

**Keeniya-Gamalage-Gehan Chaturanga De Silva** ^†,^***^a^*,** **Naomi Helsel** ^†,^***^b^*, Hirithya Sharad Jeyashangararaj^b^, Pabitra Choudhury^b*^and Sanchari Chowdhury *^b^*^*^**

^a^ Department of Chemistry, New Mexico Institute of Mining and Technology, Socorro, NM 87801, USA

^b^ Department of Chemical Engineering, New Mexico Institute of Mining and Technology, Socorro, NM 87801, USA

^†^Both authors have equal contributions

[*pabitra.choudhury@nmt.edu](mailto:*pabitra.choudhury@nmt.edu), *[sanchari.chowdhury@nmt.edu](mailto:sanchari.chowdhury@nmt.edu)


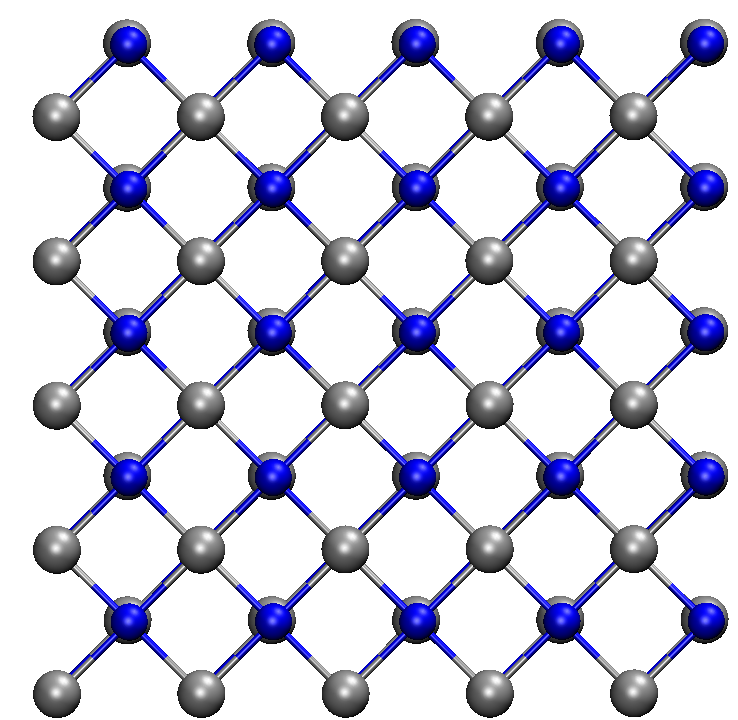

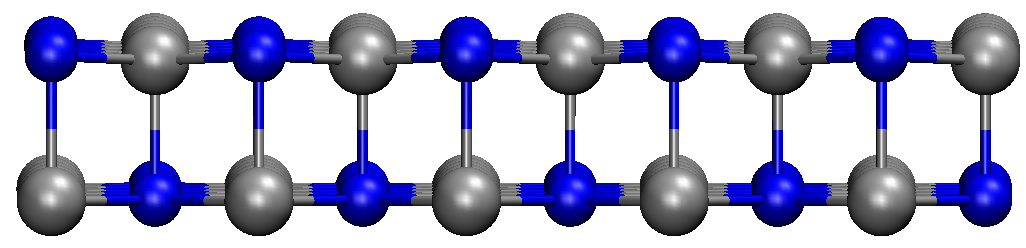


**Fig. SI.1.** Schematic representations of titanium nitride from top (left) and side view (right).

**
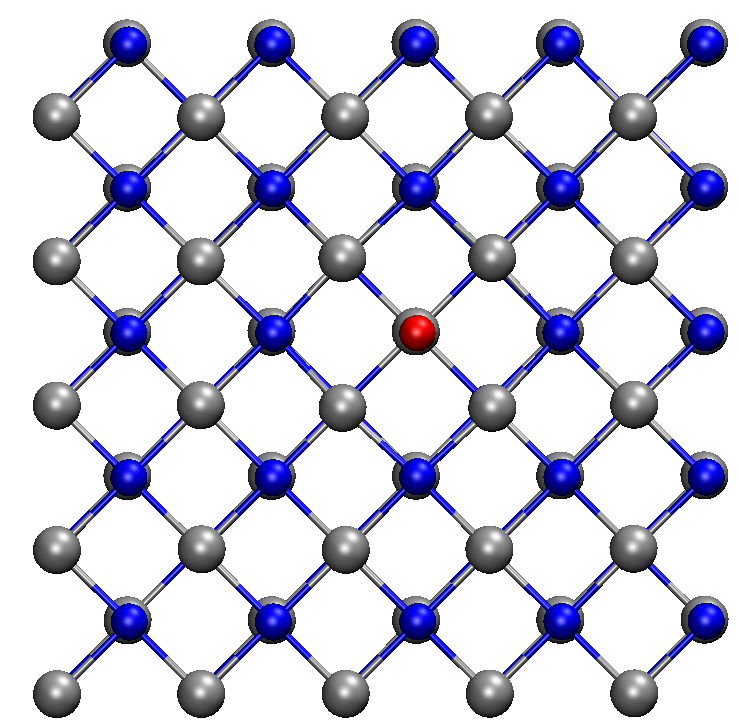
**
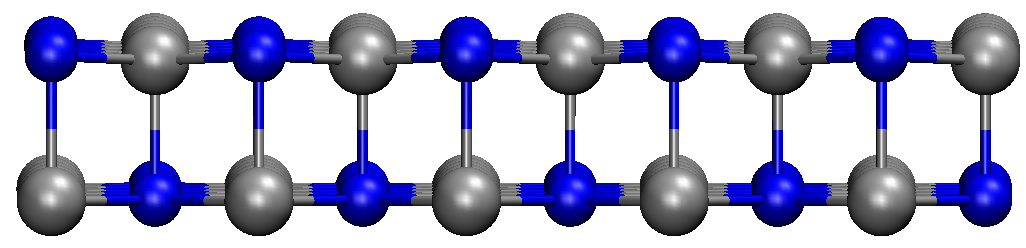


**Fig. SI.2.** Schematic representations of titanium oxynitride from top (left) and side view (right).


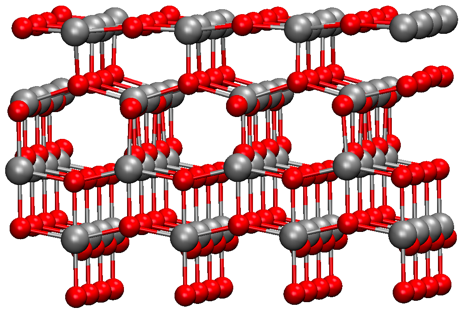

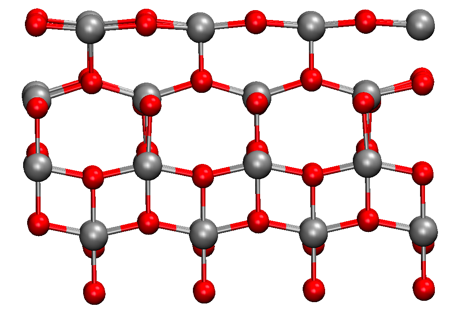

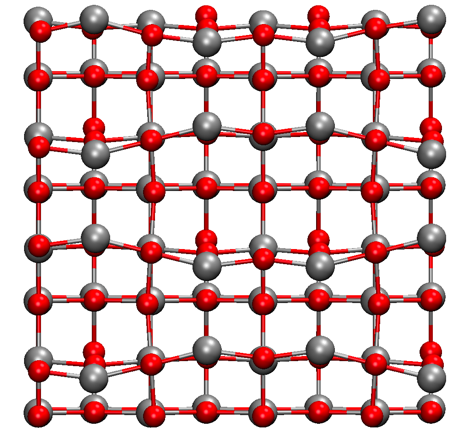


**Fig. SI.3.** Schematic representations of titanium dioxide from angled (left), side (middle), and top (right) views.


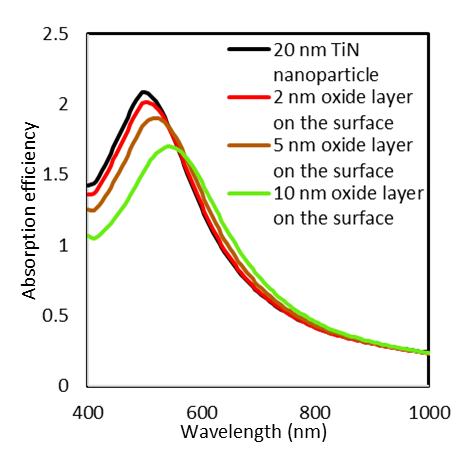


**Fig. SI.4**. Theoretically calculated absorption efficiency of 20 nm TiN nanoparticles with varied thickness of oxide layer on the surface.

**
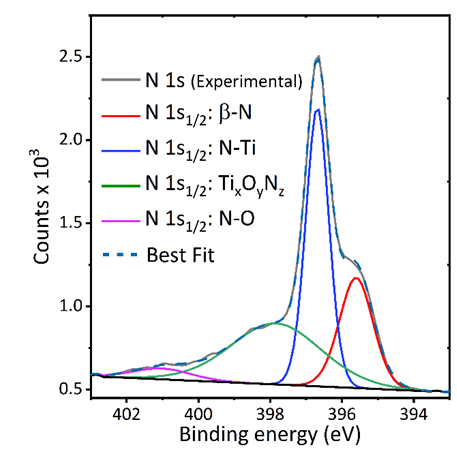
**

**Fig. SI.5 :** XPS data showing N 1S peak in TiN confirming presence of TiON, TiO_2_ and TiON on the surface.

Intensity (a.u.)

2 θ (degree)


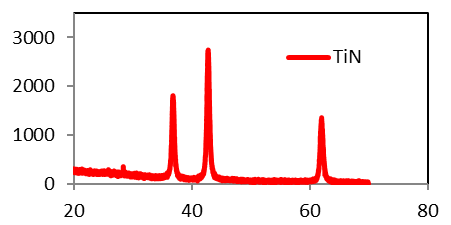


**Fig. SI.6:** X-ray diffractograms of TiN

**In-situ Photodeposition Reaction Studies using FTIR:**


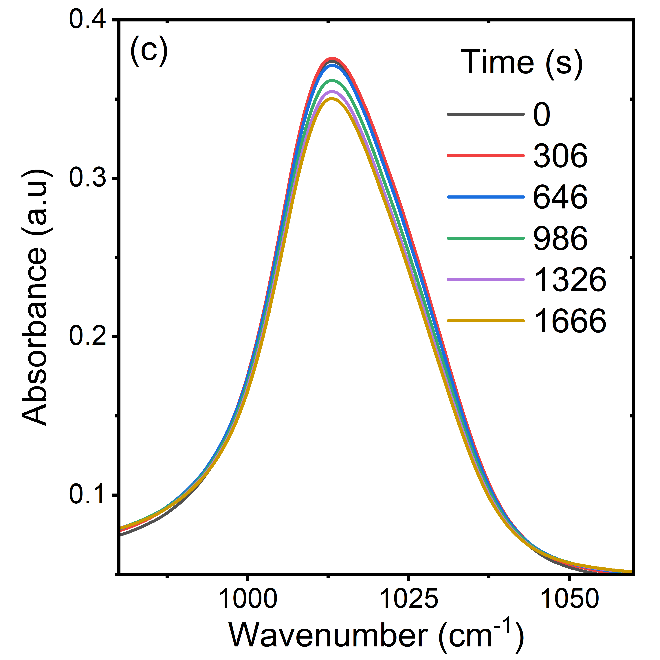

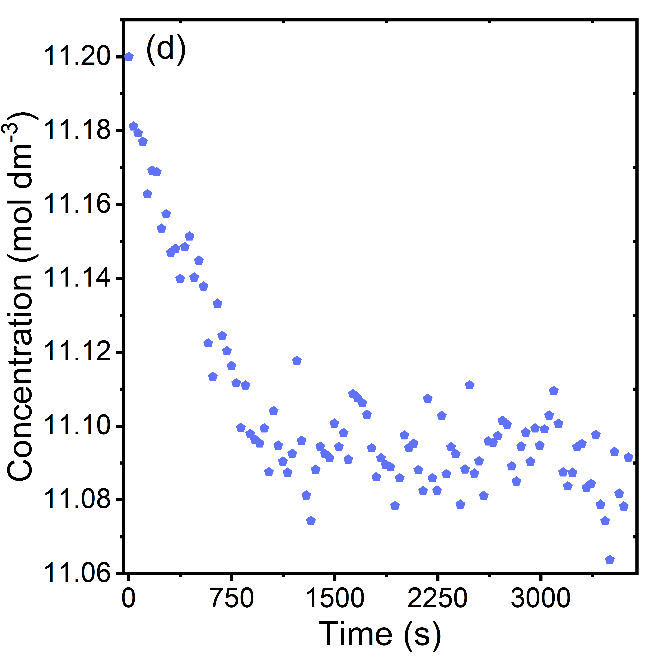

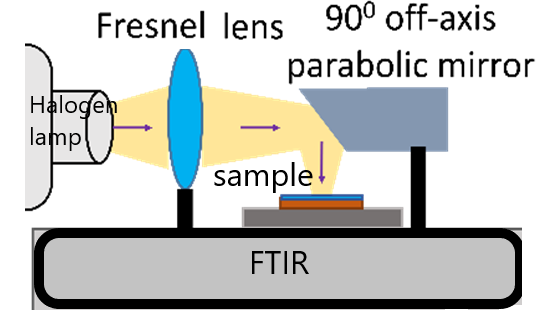


(a)


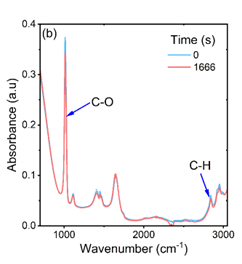


**Fig. SI. 7:** (a) Schematic of the experimental setup used for the kinetic studies using FTIR under light illumination. (b) FTIR spectrum of the reaction mixture at different times under halogen lamp intensity 192 mW cm^-2^ and the Ni^+2^ precursor mole fraction is 30% compared to TiN. (c) C-O FTIR peak variation with light irradiation time under halogen lamp intensity 192 mW cm^-2^ (d) Methanol concentration vs time plot derived from figure c.

The setup shown in Fig. SI 7 (a) was used to carry out all the kinetics studies. Kinetic studies were carried out to investigate the effect of intensity and wavelengths of light and the effect of Ni precursor mole fraction on the photodeposition reaction rate . Following are the reactions for Ni photodeposition on TiN

$Ni{Cl}_{2}+2e^{-}\to{Ni}^{0}+2{Cl}^{-}$ (1)

$CH_{3}OH+H_{2}O+6h^{+}\to CO_{2}+6h^{+}$ (2)

Based on reaction 1 and 2, reduction of Ni^+2^ can be monitored by studying the consumption of methanol. The consumption of methanol due to hole scavenging can directly correlate with the hot electrons generation. Hence, we monitor the reaction kinetics, the consumption of methanol in the reaction mixture was studied by monitoring the depletion of the FTIR peak for methanol C-O bond at ~1000 cm^-1^. Methanol shows couple of significant peaks in the FTIR spectra of the reaction mixture (Fig. SI. 7(b)). The C-O FTIR peak was selected because it is prominent in the spectra compared to the C-H bond FTIR peak. The O-H peak was never used because of the possible interference form water in the reaction mixture. Fig. SI. 7(c), shows how the C-O FTIR peak decreases with irradiation time. The data shown in the Fig. SI. 7 (b-d) were obtained for the specific trial where halogen lamp intensity is 192 mW cm^-2^ and the Ni^+2^ precursor mole fraction is 30% compared. During the entire reaction period the data were collected at every 34 s. Ones all the spectra were collected, The area under the curve for C-O peaks were converted to methanol concentration as we know the initial methanol concentration and methanol concentration Vs time was plotted (Fig. SI 7.(d)). The methanol concentration vs time plots were fitted to first order kinetics to extract rate constant.


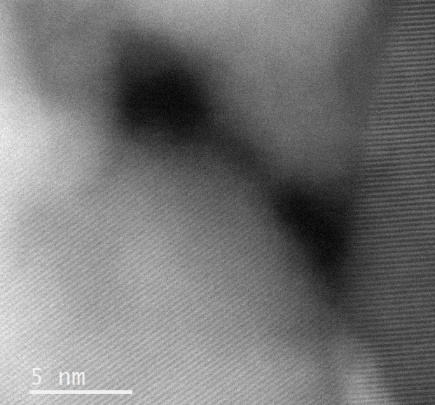


**Fig. SI 8.:** HAADF-STEM image of the control sample of TiN with Ni salt in the absence of light


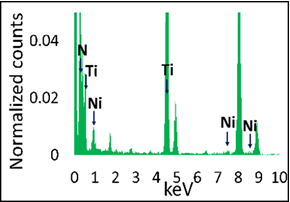


**Fig. SI.9:** JEOL Neo ARM 200CF TEM equipped with aberration correlation and Oxford Aztec EDS analysis to confirm the Ni deposition on TiN nanoparticles.


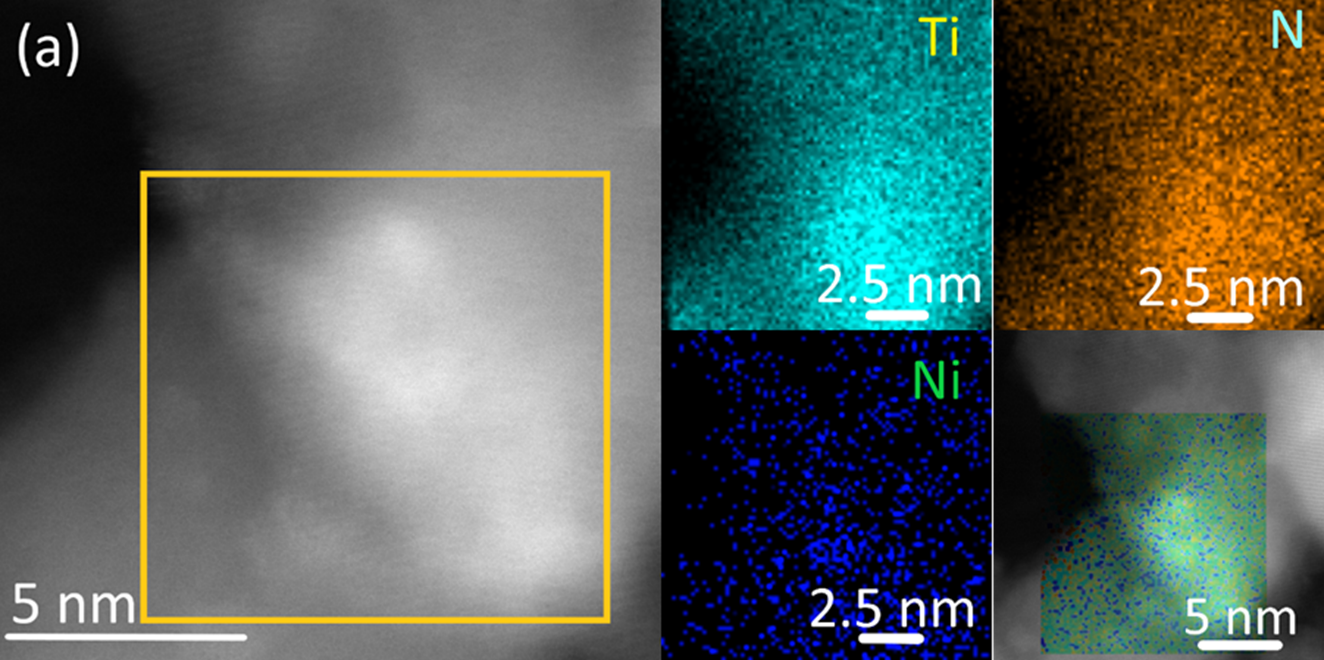


**Fig. SI. 10:** EDS mapping of Ni/TiN nanoparticle. (EDS mapping was performed for the area covered by the yellow square)

**Fig. SI.11.** Schematic representations of nickel binding sites on titanium nitride a) nitrogen-vacancy, b) nitrogen-top, c) titanium-top, and d) oxygen-top (Ti_x_O_y_N_z_) sites.

**Fig. SI.12.** Schematic representations of nickel binding sites on titanium dioxide a) Ti-bridge, b) 1-O-bridge, c) 2-O-bridge, d) 3-O-bridge, e) O-vacancy, and f) Ti-vacancy.

**Fig. SI.13.** Nickel oxidation state investigated for increasing number of hydroxide groups present on the N-vacancy Ni-TiN, Ni-TiON, and Ni-TiO_2_ surfaces.

**Table SI.1.** The electron difference after deposition for the investigated substrates calculated using: ${\Sigma Ti Charge}_{Ni\left( OH \right)_{n}+substrate}-\Sigma Ti Charge_{\begin{aligned} \left( OH \right)_{n} \\ \end{aligned}+substrate}=\Delta Ti Charge$and ${\Sigma N Charge}_{\begin{aligned} Ni\left( OH \right)_{n} \\ \end{aligned}+substrate}-\Sigma N Charge_{\begin{aligned} \left( OH \right)_{n} \\ \end{aligned}+substrate}=\Delta N Charge$(where $n$=0,1,2,4).

| **Species** | **Charge Difference on Ti Top Layer (e^-^)** | **Charge Difference on N Top Layer (e^-^)** |
| --- | --- | --- |
| Pristine Ni-TiN | 0.059 | -0.188 |
| N-vac Ni-TiN | -0.291 | -0.236 |
| N-vac Ni(OH)-TiN | 0.059 | -0.467 |
| N-vac Ni(OH)_2_-TiN | -0.068 | -0.414 |
| N-vac Ni(OH)_4_-TiN | -0.008 | -0.589 |
| Ni-TiO_x_N_y_ | 0.072 | -0.144 |
| Ni(OH)-TiO_x_N_y_ | 0.214 | -0.094 |
| Ni(OH)_2_-TiO_x_N_y_ | 0.262 | -0.003 |
| Ni-TiO_2_ | 0.066 | – |
| Ni(OH)-TiO_2_ | 0.212 | – |
| Ni(OH)_2_-TiO_2_ | 0.654 | – |
| Ni(OH)_4_-TiO_2_ | 0.286 | – |
